# Comparative analysis of essential genes in prokaryotic genomic islands

**DOI:** 10.1038/srep12561

**Published:** 2015-07-30

**Authors:** Xi Zhang, Chong Peng, Ge Zhang, Feng Gao

**Affiliations:** 1 Department of Physics, Tianjin University, Tianjin 300072, China; 2 Key Laboratory of Systems Bioengineering (Ministry of Education), Tianjin University, Tianjin 300072, China; 3 SynBio Research Platform, Collaborative Innovation Center of Chemical Science and Engineering, Tianjin 300072, China

## Abstract

Essential genes are thought to encode proteins that carry out the basic functions to sustain a cellular life, and genomic islands (GIs) usually contain clusters of horizontally transferred genes. It has been assumed that essential genes are not likely to be located in GIs, but systematical analysis of essential genes in GIs has not been explored before. Here, we have analyzed the essential genes in 28 prokaryotes by statistical method and reached a conclusion that essential genes in GIs are significantly fewer than those outside GIs. The function of 362 essential genes found in GIs has been explored further by BLAST against the Virulence Factor Database (VFDB) and the phage/prophage sequence database of PHAge Search Tool (PHAST). Consequently, 64 and 60 eligible essential genes are found to share the sequence similarity with the virulence factors and phage/prophages-related genes, respectively. Meanwhile, we find several toxin-related proteins and repressors encoded by these essential genes in GIs. The comparative analysis of essential genes in genomic islands will not only shed new light on the development of the prediction algorithm of essential genes, but also give a clue to detect the functionality of essential genes in genomic islands.

Extensive studies have been carried out for searching the minimal set of genes that can sustain a bacterial cell under ideal conditions^1^,^2^. The minimal set of genes are usually referred to as essential genes, which encode the basic functions to sustain a cellular life involving DNA replication, transcription, RNA processing, amino acyl-RNA formation and protein folding^3^. In recent years, it has been argued that whether exists a universal minimal genome, what functions of essential genes are universal to sustain a cell life and what are essential only for particular cell types^4^. Although there is no general consensus about what a universal minimal genome would be, it is believed that controllable minimal cells could be created with the development of synthetic biology^5^. With the high throughput techniques springing up, essential genes in different organisms could be detected on a genome-wide scale. For example, essential genes in *H. pylori*, *A. baylyi* ADP1 and *S. aureus* have been identified by microarray tracking of transposon mutants (MATT), single-gene deletion mutants and antisense RNA inhibition, respectively^6^,^7^,^8^. However, it is not safe to say essential genes detected by experimental methods must be credible and undisputed, because essential genes required for growth on different media are experimentally detected and different methods may cause different detection results^9^. For example, the saturation transposon mutagenesis methods may mistakenly choose the genes which only slow down the growth^4^. The transposon bombardment can yield slightly different essential gene sets as the method is not entirely random^5^. Inactivating individual genes may miss essential genes which present in more than one copy^10^,^11^.

A new view has been raised in recent years, which argues that combining the experimental with computational methods to determine which of these identified genes are really essential^4^. The early attempt to identify essential genes in a computational way is using the comparative methods^12^. Those shared between organisms are assumed to be essential, but this method is greatly influenced by the homology. For example, if the evolutionary distance is too far, it can greatly decrease the number of shared genes identified by comparative genomics. Therefore, it is better to firstly identify the essential genes in computational way and then testify the essentiality by different experimental methods. Recently, the essential genome of *P. aeruginosa* has been established with statistical precision by a Monte Carlo simulation method^13^. Although it is a difficult and time consuming task to decide which of these identified genes are really required for growth or form the universal minimal genome, combining the reliable computational with experimental methods to determine essential genes between a huge set of species would open opportunities for better understanding the essence of life. It has been proposed that essential genes existing only within a specific evolutionary lineage are presumed to be crucial for the living process^5^. Besides, novel essential genes predicted by a comparative analysis of the phylogenetically related organisms can give a clue to identify the potential antimicrobials targets^14^.

A comprehensive and periodically updated database of essential genes (DEG) was published in 2004^15^, which includes nearly all published essential genes, and there are 34 bacterial and archaeal records corresponding to 30 organisms in total^3^,^16^. Some studies focusing on the enrichment analysis of essential genes versus non-essential genes have been performed before^17^,^18^. More significant comparison results have been obtained based on this database recently. For example, enzymes are enriched in essential genes across multiple bacterial genera^19^, essential genes which preferentially reside in the leading strand are more evolutionarily conserved compared with non-essential ones^20^, and proteins encoded by essential genes are enriched in internal sites and have a lower proportion in cell envelope versus the non-essential ones^21^. Besides, based on the developing studies, essential genes prediction models and tools spring up^22^,^23^,^24^. Other studies on enrichment analysis can also give a clue to our current work^25^,^26^,^27^.

Genes that horizontally transfer to the host may form a novel pathway essential for cell survival or encode proteins more efficient than those originally produced by the old host genes^28^. However, essential genes with horizontally acquired regions across multiple genera have not been systematically examined, so statistical analysis of the essential genes in GIs is on the agenda. In this study, we focus on the essential genes in genomic islands (GIs). GIs commonly contain clusters of horizontally transferred genes. These genes usually encode the functions of toxins and adhesins, type III secretion systems and iron uptake which are regarded as an improvement for the pathogens to cause disease and survive in the bacteria^29^,^30^. Besides, mobile elements are frequently found in GIs, such as integrases, transposases, insertion sequence elements and some particular genes which encode cell surface proteins, virulence factors, host interactions proteins, DNA-binding proteins and phage-related proteins^31^,^32^,^33^. Because of these characters of GIs and the necessity of essential genes, it has been believed that fewer essential genes are located in the GIs than those outside the GIs. The functions of essential genes found in GIs should be further studied. Knowledge of essential genes in genomic islands is of great importance, because it not only gives a better understanding of what the gene set of universal minimal genome would be, but also helps a lot in the recognition of new essential genes.

## Results and Discussion

**Figure 1:**
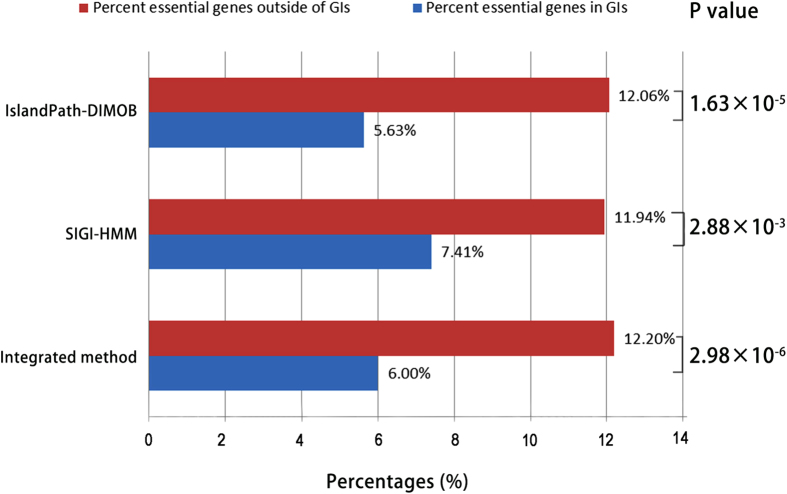
Average percentages of essential genes located in GIs and out of GIs. The three methods used to detect GIs are listed in the vertical axis. The *P* values from Student’s *t* test are also displayed in the figure.

**Figure 2:**
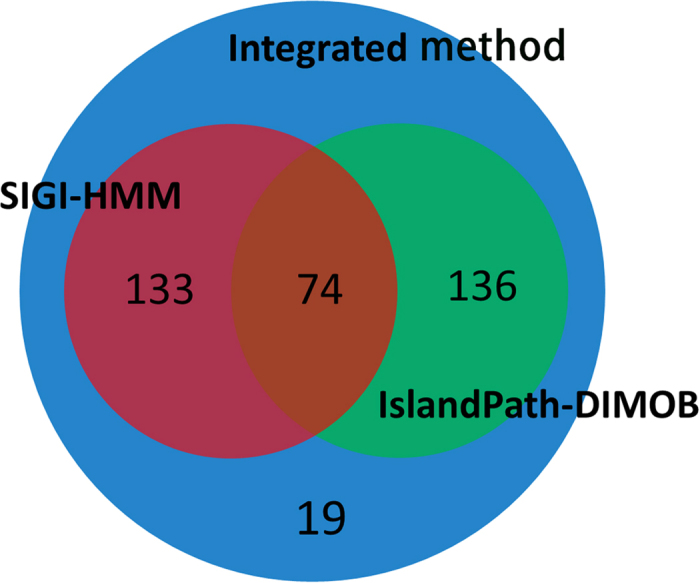
The Venn diagrams for the number distribution of essential genes located in GIs. The three circles represent IslandPath-DIMOB, SIGI-HMM and Integrated method, respectively.

**Figure 3:**
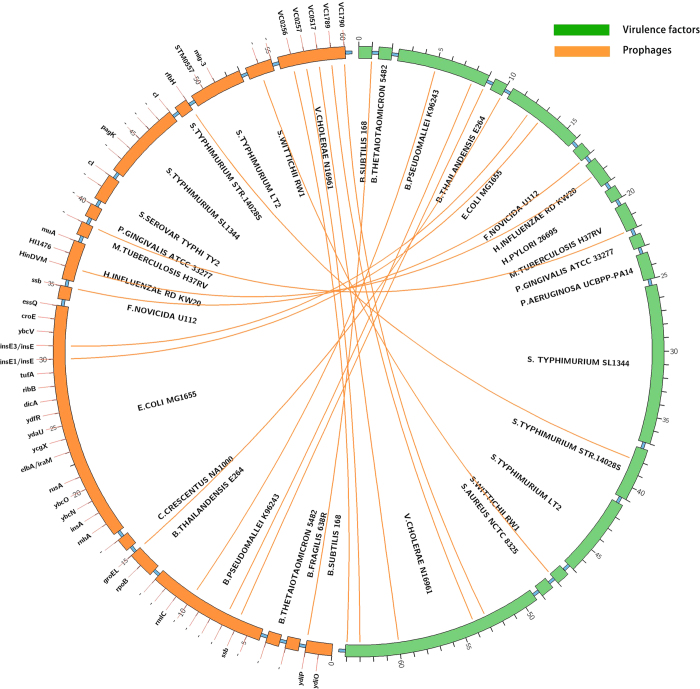
The circos plot of virulence factors (green) and prophages (yellow) that share similar sequences. Each word in the inner band is the name of the identified organism, each word outside the left half band shows the name of the gene (the character ‘-’ means ‘unknown gene’). Each number around the circle shows the serial number of selected gene in the dataset of virulence factors and prophages.

We used a dataset of 27 bacteria and 1 archaeon from the DEG database^16^, and identified the genomic islands (GIs) among the 28 prokaryotic organisms. A web resource called IslandViewer^34^,^35^, which has been updated to IslandViewer 3 recently 35, has been used to identify genomic islands in the current study. IslandViewer has integrated three credible and representative methods, i.e., IslandPath-DIMOB^36^, SIGI-HMM^33^ and Integrated method^37^, which makes it popular and convenient for the researchers to compare the differences of alternative methods. It should be mentioned that Integrated method which is thought to have a higher overall accuracy and lower sensitivity contains two sequence composition methods (IslandPath-DIMOB and SIGI-HMM) and a comparative methods named IslandPick. However, IslandPick^38^ is a method with higher accuracy but lower sensitivity, which detects less quantity of GIs, so it is unwisely used as a singular method. *Campylobacter jejuni* and *Mycoplasma genitalium* were not selected for their lack of genomic islands. To comprehensively quantify the occurrence of essential genes in GIs, a systematical examination has been conducted, and the results are displayed in Table 1 and Fig. 1. In Fig. 2, Venn diagram shows the overlap of essential genes in GIs predicted by the three methods (IslandPath-DIMOB, SIGI-HMM and Integrated method). We found that there are 210, 207 and 362 essential genes in the GIs identified by the three GI detection methods, respectively. As for *Pseudomonas aeruginosa* PAO1, *Shewanella oneidensis* MR-1, *Staphylococcus aureus* N315, *Streptococcus pneumonia* and *Streptococcus sanguinis*, no essential genes were found in GIs. Owing to the lowest *p* value and adequate data, the dataset of 362 essential genes are explored further. To comprehensively identify functions of these essential genes, BLAST similarity search^39^ has been carried out against the database of the Virulence Factor Database (VFDB)^40^ and the phage/prophage sequence database of PHAge Search Tool (PHAST)^41^. Consequently, 64 and 60 eligible essential genes have been found sharing the sequence similarity with the virulence factors and phage/prophages-related genes, respectively. Meanwhile, we find several toxin-related proteins and repressors encoded by these essential genes in GIs. In Fig. 3, a similar association between the dataset of the virulence factors and prophages is created by circos^42^. Six tables (S1-S6) with details of essential genes located in genomic islands are available at http://tubic.tju.edu.cn/eg-gi/. The dataset of 362 essential genes across 28 prokaryotic organisms in GIs confirmed by Integrated method is listed in Table S1, including the accession number of DEG database^16^, the gene name, the GI number, COG contents, function class, function description and the name of the organism. Similar datasets (Table S2 and Table S3) for other GIs prediction methods (IslandPath-DIMOB and SIGI-HMM) are also available online. Tables S4-S6 list 64 eligible essential genes sharing the sequence similarity with virulence factors, 60 eligible essential genes sharing the sequence similarity with prophages and 17 eligible essential genes sharing the common sequence similarity between virulence factors and prophages, respectively.

### Essential genes in the GIs are statistically fewer than those outside the GIs

We first submitted the genome sequences of the 28 organisms to IslandViewer, and obtained three genomic islands datasets by the three methods: IslandPath-DIMOB, SIGI-HMM and Integrated method. Based on a dataset of 10,789 essential genes of bacteria and 519 essential genes of archaeon from the DEG database, the proportions of essential genes in and out of GIs can be studied, respectively. The statistical result is displayed in Fig. 1. IslandPath-DOMB, which is defined as a more strict and more precise method, is based on sequence composition method, both dinucleotide bias and the presence of one or more mobility genes in the genes region can be regarded as GIs^36^. From Fig. 1, by means of IslandPath-DOMB, the average percentages of essential genes in and out of GIs are 5.63% and 12.06%, respectively, and the Student’s *t* test shows that the difference is statistically significant (*p *= 1.63 × 10^−5^). SIGI-HMM, which is usually thought to have the highest overall accuracy, is based on an analysis of the codon usage which removes ribosomal regions^33^. By means of SIGI-HMM, the average percentages of essential genes in and out of GIs are 7.41% and 11.94%, respectively, and the Student’s *t* test shows that the difference is statistically significant (*p *= 2.88 × 10^−3^). Integrated method, which has higher overall accuracy and lower sensitivity, integrates IslandPath-DOMB, SIGI-HMM and IslandPick. IslandPick is based on the comparative methods and can be run using manually selected comparison genomes instead of default genomes^38^. By means of the Integrated method, the average percentages of essential genes in and out of GIs are 6.0% and 12.20%, respectively, and the Student’s *t* test shows that the difference is statistically significant (*p *= 2.98 × 10^−6^). It should be mentioned that some GIs could not be identified, if they share the similar sequence composition with host genomes or may be forced to ameliorate themselves over time^43^, and some predicted GIs may appear as false-positive predictions like those that contain highly expressed genes encoding ribosomal proteins^44^, if sequence composition bias is used as the only criterion to identify GIs. In this study, there are 38 and 32 essential genes encoding ribosomal proteins in the identified genomic islands of *Bacteroides fragilis* 638R and *Salmonella enterica* serovar Typhi Ty2, respectively, which is unusual because ribosomal proteins encoded by the clusters of essential genes are vital to cellular life. In these cases, we would use IslandPick to run a custom analysis to rule out potential false positive predictions. Because we suspect IslandPath-DIMOB predicts these as GIs due to a dinucleotide bias in the region and one hypothetical protein matched a HMMER^45^ scan for mobility genes. All the corresponding *p* values are less than 0.05, which means that the differences are statistically significant. The conclusion that essential genes in GIs are rarer is statistically obvious, whatever predicting methods we choose.

**Figure 4:**
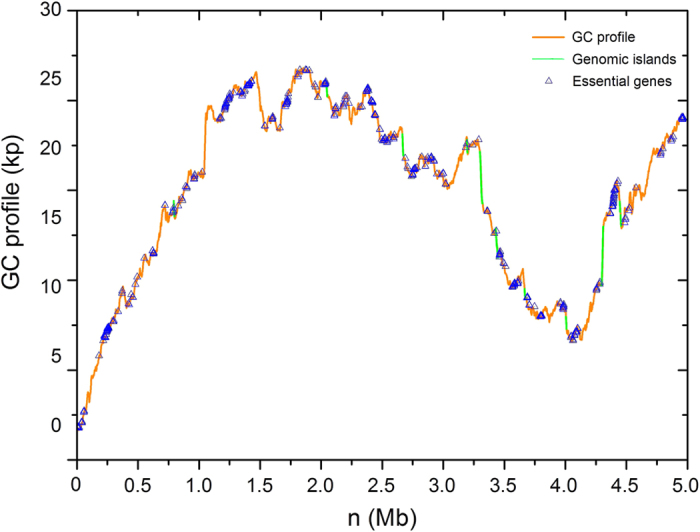
GC profile for the genome of *Shewanella oneidensis* MR-1. The green line segments represent GIs. The blue triangles represent essential genes.

For convenience, the information of the organisms is listed in Table 1. From the table we can find that essential genes in the GIs have a lower proportion compared with the complementary set of essential genes. The percentage of essential genes in GIs is much lower than that outside GIs. Such results verify the assumption that essential genes are not likely to be located in GIs. Essential genes usually encode the proteins that carry out the basic functions to sustain a cellular life, such as DNA replication, transcription, RNA processing, amino acyl-RNA formation and protein folding^3^. However, GIs which usually encode the functions of toxins and adhesins, type III secretion systems and iron uptake functions, are regarded as an improvement for the pathogens to cause disease and survive in the bacteria^29^,^30^. To clearly demonstrate that essential genes are disproportionately in GIs versus those outside GIs, GC-Profile^46^, an online service for visualizing and analyzing the variation of GC content, has also been applied, and the results are displayed in Fig. 4. From the figure, the essential genes (blue) are disproportionately located in GIs (green) versus those outside GIs (brown) in *Shewanella oneidensis* MR-1.

### Functional analysis of essential genes in GIs

There is a thorny challenge in the area of bacterial homology detection that can better understand the genetic repertoire of different bacterial lifestyles^47^. What deters most of researchers is how to develop a reliable method for protein homology detection. In recent years, based on a density parameter that has been widely neglected, *Röttger et al*. brought out an artful protein homology detection method which enlightens our work most^47^. Similarly, the essentiality of essential genes is crucial but hard to estimate without a consolidated standard. It is not easy to say essential genes detected by experimental methods must be credible and undisputed, because essential genes required for growth on different media are experimentally detected and different methods may cause different detection results^9^. Thus, combining the reliable computational with experimental methods to determine essential genes between a huge set of species would open opportunities for better understanding the essence of life.

Here, after discussing the impact caused by different experimental methods of essential genes and prediction algorithm of genomic islands, a conclusion that essential genes are statistically fewer in the GIs versus those outside GIs has been reached. Considering the lowest *p* value and adequate data, the dataset of 362 essential genes are chosen to have a further analysis. Two datasets which contain five Gram-positive bacteria and twenty one Gram-negative bacteria are chosen to have a further analysis of essential genes in genomic islands. For the dataset of Gram-positive bacteria, the average percentages of essential genes in and out of GIs are 3.51% and 10.73% (with standard deviation 0.074 and 0.0233), respectively. However, in regard to the dataset of Gram-negative bacteria, the average percentages of essential genes in and out of GIs are 8.01% and 14.44% (with standard deviation 0.075 and 0.095), respectively. It demonstrates that the average percentages of essential genes located in GIs of Gram-positive bacteria are lower in comparison to the Gram-negative ones, which may give referenced values in the related analysis. We further study the essential genes in GIs of two Gram-positive bacteria which are newly updated in DEG database. It is found that the average percentages of essential genes in GIs are 2.36% and 3.75% for *Streptococcus pyogenes* MGAS5005 and *Streptococcus pyogenes* NZ131, respectively. The average percentages of essential genes outside GIs are 12.89% and 14.69% for *S. pyogenes* MGAS5005 and *S. pyogenes* NZ131, respectively. It seems that the percentages of essential genes in and outside GIs are in a relatively stable proportion for both Gram-positive bacteria and Gram-negative bacteria, which can be further proved with more bacterial essential genes detected.

To have a further analysis of the homologous proteins of essential genes, BLAST similarity search (BLAST score >100, E value <10-e5) has been carried out against the Virulence Factor Database (VFDB) and the phage/prophage sequence database of PHAge Search Tool (PHAST). Consequently, 64 and 60 eligible essential genes share the sequence similarity with the virulence factors and phage/prophages-related genes, respectively. The dataset of virulence factors and prophages is displayed in Fig. 3. From the figure, a similar association between the virulence factors (green) and prophages (yellow) is created by circos^42^. The sporadic yellow lines show that 17 genes share the common sequence similarity between virulence factors and prophages. The outer green band represents seventeen organisms (*B. subtilis* 168, *B. thetaiotaomicron* VPI-5482, *B. pseudomallei* K96243, *B. thailandensis* E264, *E. coli* MG1655, *F. novicida* U112, *H. influenzae* Rd KW20, *H. pylori* 26695, *M. tuberculosis* H37Rv, *P. gingivalis* ATCC 33277, *P. aeruginosa* UCBPP-PA14, *S. Typhimurium* SL1344, *S. Typhimurium* str. 14028S, *S. typhimurium* LT2, *S. wittichii* RW1, *S. aureus* NCTC 8325, *V. cholerae* N16961) in clockwise, respectively. The outer yellow band represents the seventeen organisms (*B. subtilis* 168, *B. fragilis* 638R, *B. thetaiotaomicron* VPI-5482, *B. pseudomallei* K96243, *B. thailandensis* E264, *C. crescentus* NA1000, *E. coli* MG1655, *F. novicida* U112, *H. influenzae* Rd KW20, *M. tuberculosis* H37Rv, *P. gingivalis* ATCC 33277, *S. Typhi* Ty2, *S. Typhimurium* SL1344, *S. Typhimurium* str. 14028S, *S. typhimurium* LT2, *S. wittichii* RW1, *V. cholerae* N16961) in clockwise, respectively. According to the size of the outer band, we can clearly make out the proportion of essential genes in the datasets of virulence factors and prophages among multiple organisms.

We further analyze the function of essential genes in the datasets of virulence factors and prophages. Firstly, it is assumed that the horizontally transferred genes can block the transposition insertions. The restriction of transposon reduces the insertion at these sites, so some ‘essential genes’ might not be really ‘required’. For example, few insertions can be found within the essential genes in pathogenicity island of *S. Typhimurium* SL1344, among which, *spiC, sseA and ssaHIJST* encode the function of type III secretion system apparatus^48^. In this case, we also found the essential genes (*PA14_42540* and *pscO*), which encode the function of type III secretion system in pathogenicity island of *P. aeruginosa* UCBPP-PA14 from the dataset of virulence factors. Secondly, it is believed that stable toxin proteins outlive unstable antitoxin proteins and stable toxin proteins intend to protect their own survival and attack other foreign DNA^28^. In this case, toxin may be not really needed for the host. Once they transfer to the host, they will try their best to integrate into their host’s regulatory network^28^. From the dataset of virulence factors, we found five essential genes (*BPSL1665*, *BPSL1664*, *VC0837, VC0836, VC0834*) which encode the function of toxin-related protein, are located in the pathogenicity island of *B. pseudomallei* K96243 and *V. cholerae* N16961. Thirdly, it is noteworthy that the horizontally transferred genes which encode the repressor maintaining the lysogenic state of prophage and preventing transcription of early lytic genes may be not always required for cellular life. Because the repressor encoded by these genes is required for continual viability as long as the rest of the phages remain intact^28^. From the dataset of prophages, we find four essential genes (*b1145*, *b1570*, *t4337*, *SL1344_2708*) which encode repressor protein located in the pathogenicity island of *E. coli* MG1655, *S. Typhi* Ty2 and *S. Typhimurium* SL1344. These findings can be a reminder that the explanation of essential genes should be carefully made. Because of too many external factors, classifying the genes of different pathogenicity lifestyles into train-specific or lifestyle-specific in a computational way is limited^49^. The mechanism of horizontally transferred genes in a cellular life should be further studied especially combining experimental method with computational method to avoid choosing those not actually required for growth.

## Conclusion

After minimizing the impact caused by different experimental methods of essential genes and GI detection methods, our results show that fewer essential genes are located in the GIs versus those outside GIs. Based on the database of VFDB and PHAST, we identify 64 and 60 essential genes sharing the sequence similarity with virulence factors and phage/prophages-related genes, respectively. Meanwhile, five toxin-related essential genes and four essential genes encoding for repressors have been detected in pathogenic GIs. The mechanism of horizontally transferred genes in a cellular life should be further studied especially combining experimental method with computational method. The comparative analysis of essential genes in genomic islands will not only shed new light on the development of the prediction algorithm of essential genes, but also give a clue to detect the functionality of essential genes in genomic islands. Although it is a difficult and time consuming task to decide which of these identified genes are really required for growth or forming the universal minimal genome, we believe in the future not far away, combining reliable computational methods with experimental methods to determine essential genes between a huge set of species would open opportunities for better understanding the essence of life.

## Materials and Methods

### Bioinformatics Databases

A comprehensive and periodically updated database of essential genes (DEG) was published in 2004, which includes nearly all published essential genes^3^,^16^. There are 34 bacterial records corresponding to 30 organisms in the database in total and 28 sets of data are selected in our study. *Campylobacter jejuni* and *Mycoplasma genitalium* are excluded for their lack of GIs. As for the organisms with multiple records, the one with the most convincing experimental methods has been chosen. So *Escherichia coli* MG1655 I, *Mycobacterium tuberculosis* H37Rv III and *Salmonella enterica* serovar Typhi Ty2 have been chosen among the multiple records of the database. The information of the organisms is displayed in Table 1.

A basic local alignment search tool (BLAST)^39^ similarity search was carried out against the database of VFDB^40^ (a web-based database of virulence factors, available at http://www.mgc.ac.cn/VFs/) and PHAST^41^ (a tool for finding the phage, available at http://phast.wishartlab.com). A similar association between the dataset of virulence factors and prophages is created by circos (a software package of visualizing data and the data can be showed in a circular layout, available at http://mkweb.bcgsc.ca/circos) and the result is displayed in Fig. 3.

### Software Tools

IslandViewer (available at http://www.pathogenomics.sfu.ca/islandviewer) is an integrated tool for identifying and visualizing genomic islands, which integrates two sequence composition GI prediction methods SIGI-HMM and IslandPath-DIMOB, and a single comparative GI prediction method IslandPick. GIs are detected by the three methods: IslandPath-DIMOB^36^ detects the GIs with a region of 8 or more ORFs with dinucleotide bias plus the presence of one or more mobility genes (a more specific method of GI detection); SIGI-HMM^33^ is usually thought to have the highest overall accuracy for sequence composition method, and is based on an analysis of codon usage which removes ribosomal regions; IslandPick is based on a comparative method and can be run by using manually selected comparison genomes instead of default genomes^38^. It should be noteworthy that the number of essential genes outside GIs is in majority, so we normalize the proportion of essential genes inside and outside the islands. The percentage of essential genes in and out of GIs is demonstrated in Table 1 (Y, Z). To clearly show the essential genes are disproportionately located in GIs versus those outside GIs, GC-Profile which is an online service for visualizing and analyzing the variation of GC content is applied (available at http://tubic.tju.edu.cn/GC-Profile/), and the result is displayed in Fig. 4. Venn diagram plotted by R package shows the overlap of essential genes in GIs predicted by three methods: IslandPath-DIMOB, SIGI-HMM and Integrated method, whose results are displayed in Fig. 2.

### Test Method

The Student’s *t* test has been performed to test the significance of difference between the proportions of essential genes in and out of GIs. The Student’s *t* test is a method for testing whether the means of two groups are statistically different from each other. *P* value less than 0.05 is considered statistically significant.

## Additional Information

**How to cite this article**: Zhang, X. *et al*. Comparative analysis of essential genes in prokaryotic genomic islands. *Sci. Rep*. **5**, 12561; doi: 10.1038/srep12561 (2015).

## Figures and Tables

**Table 1:** The information of the organisms used in the current study.

Organism	RefSeq	Group^a^	No.eg^b^	IslandPath-DIMOB(%)^c^			SIGI-HMM(%)^c^			Integrated method (%)^c^		
*A. baylyi* ADP1	NC_005966	Bacteria(−)	499	0.4	5.88	15.18	0.6	7.5	15.18	1	6.41	15.3
*B. subtilis* 168	NC_000964	Bacteria(+)	271	0	0	6.97	1.11	2.61	6.6	1.11	0.89	6.98
*B. fragilis* 638R	NC_016776	Bacteria(−)	547	3.84	11.67	12.8	8.59	17.03	12.46	10.06	13.96	12.63
*B. thetaiotaomicron* VPI-5482	NC_004663	Bacteria(−)	325	2.46	3.15	7.01	2.15	2.73	7.03	5.23	3.46	7.18
*B. pseudomallei* K96243	NC_006350	Bacteria(−)	505	6.34	15.46	14.82	4.16	9.86	15.2	6.93	8.37	15.77
*B. thailandensis* E264	NC_007651	Bacteria(−)	406	3.2	4.98	13.03	0	0	12.7	3.2	4.68	13.11
*C. crescentus* NA1000	NC_011916	Bacteria(−)	480	1.25	4.55	12.63	0.83	3.64	12.61	1.88	5.56	12.65
*E. coli* MG1655	NC_000913	Bacteria(−)	609	5.26	15.53	14.67	5.09	10.44	15.04	9.03	14.55	14.73
*F. novicida* U112	NC_008601	Bacteria(−)	392	1.02	12.5	23	0.26	20	22.81	1.02	8.33	23.22
*H. influenzae* Rd KW20	NC_000907	Bacteria(−)	642	1.25	44.44	39.82	0.62	17.39	40.2	2.34	32.61	40.09
*H. pylori* 26695	NC_000915	Bacteria(−)	323	1.24	8.89	22.4	0	−	−	1.24	8.89	22.4
*M. maripaludis* S2	NC_005791	Archaeon	519	0.58	7.69	30.66	0	−	−	0.58	7.69	30.66
*M. tuberculosis* H37Rv	NC_000962	Bacteria(−)	687	1.6	8.53	17.9	0.58	40	17.53	2.18	11.36	17.81
*M. pulmonis* UAB CTIP	NC_002771	Mycoplasmas	310	0.65	9.52	40.47	0	−	−	0.65	9.52	40.47
*P. aeruginosa* PAO1	NC_010729	Bacteria(−)	463	2.16	6.9	23.3	0	0	22.41	2.16	6.67	23.36
*P. gingivalis* ATCC 33277	NC_002516	Bacteria(−)	117	0	0	2.12	0	0	2.13	0	0	2.16
*P. aeruginosa* UCBPP-PA14	NC_008463	Bacteria(−)	335	0	0	5.83	0	0	5.7	1.19	1.69	5.85
*S. aureus* N315	NC_004631	Bacteria(+)	358	0.84	0.86	8.86	0	−	−	1.12	0.69	9.39
*S. oneidensis* MR-1	NC_016810	Bacteria(−)	353	2.83	7.3	7.96	9.63	11.6	7.68	10.48	9.54	7.79
*S. pneumoniae*	NC_016856	Bacteria(+)	105	1.91	0.73	2.04	13.33	4.13	1.83	13.33	2.57	1.91
*S. sanguinis* SK36	NC_003197	Bacteria(+)	230	1.74	1.15	5.51	6.52	5	5.18	7.83	4.04	5.29
*S. Typhi* Ty2	NC_004347	Bacteria(−)	403	0	−	−	0	0	10.07	0	0	10.07
*S. Typhimurium* SL1344	NC_009511	Bacteria(−)	535	2.24	6.9	11.18	0.19	1.59	11.16	2.24	5.74	11.27
*S. Typhimurium* str. 14028S	NC_002745	Bacteria(−)	302	0	0	12.09	0	0	11.84	0	0	12.19
*S. typhimurium* LT2	NC_007795	Bacteria(−)	351	1.14	16.67	12.65	0	0	12.69	1.14	16.67	12.65
*S. wittichii* RW1	NC_003028	Bacteria(−)	244	0	0	11.84	0	−	−	0	0	11.84
*S. aureus* NCTC 8325	NC_009009	Bacteria(+)	218	0	0	9.66	0	0	9.91	0	0	9.97
*V. cholerae* N16961	NC_002505	Bacteria(−)	779	2.44	18.81	31.24	2.31	17.82	31.28	3.08	19.05	31.35

^a^Bacteria(+), Gram-positive bacteria; Bacteria(−), Gram-negative bacteria.

^b^Number of essential genes of the organism.

^c^The dataset classified by IslandPath-DIMOB (or SIGI-HMM, Integrated method) contain three numbers (%): X, Y, Z. X% is the percentage of essential genes which located in the GIs detected by the IslandPath-DIMOB (or SIGI-HMM, Integrated method) among the total essential genes of the organism. Y% is the percentage of essential genes in GIs. Z% is the percentage of essential genes outside GIs. The character ‘–’ in the column of IslandPath-DIMOB or SIGI-HMM means no genomic island is detected by the corresponding method.
